# The impact of social organizations on HIV/AIDS prevention knowledge among migrants in Hefei, China

**DOI:** 10.1186/s12992-018-0359-4

**Published:** 2018-04-25

**Authors:** Wenting Wang, Ren Chen, Ying Ma, Xuehui Sun, Xia Qin, Zhi Hu

**Affiliations:** 1 School of Health Service Management, Anhui Medical University, No.81 Meishan Road, Hefei, 230032 China; 2 Party Committee Office, Hangzhou First People’s Hospital, No.261 Huansha Road, Hangzhou, 310006 China

**Keywords:** Migrants, Social organizations, HIV/AIDS, Political party, Alumni association

## Abstract

**Background:**

There is a growing recognition of the need to provide HIV/AIDS prevention and care to migrant workers. Social involvement, a type of social capital, is considered a ‘critical enabler’ of effective HIV/AIDS prevention. Designated participation in formal community groups by the government (e.g., political parties) and informal, voluntary local networks by NGOs (e.g., alumni association, cultural & sports club) play different roles in HIV prevention. The objective of this study is to assess the impact of different types of social organizations on HIV/AIDS prevention knowledge among migrant workers.

**Methods:**

A cross-sectional study of 758 migrants was conducted in Hefei, Anhui Province, China. Data were collected through a self-reported questionnaire. Logistic regression was used to assess associations between different social organizations and HIV/AIDS prevention.

**Results:**

Migrants who participated in social organizations had a higher awareness of HIV/AIDS knowledge than migrants who do not participate in social organizations. Higher levels of HIV/AIDS knowledge is associated with positive HIV/AIDS behaviors for people who attended political parties (odds ratio [OR] = 3.49, 95% CI: 1.22-9.99). This effect is not significant for alumni association. For both political parties and alumni association members (OR = 0.19, 95% CI: 0.06-0.66, OR = 0.20, 95% CI: 0.08-0.61, respectively), people who exhibited higher levels of HIV/AIDS knowledge had more negative attitudes than those with less knowledge.

**Conclusion:**

Social organizations play an important role in improving HIV/AIDS knowledge and behavior in migrants, providing a great opportunity for HIV/AIDS prevention.

## Introduction

The economy in China has developed rapidly over the past several years, and as a result there is an increase in the number of migrants as sources of social capital. Large-scale population migration has unique economic, social and demographic impacts, which may influence the risk of HIV transmission [1]. In China, there were an estimated 780,000 people living with HIV/AIDS (PLWHAs), and migrants accounted for 21.0% of PLWHAs [2]. Many factors including limiting familial and social network support, forcing migrants into physically demanding jobs and poor housing and living conditions limit migrants’ access to health care and health information, which put migrants at higher risk of HIV infection [3]. Migrants are an important vector for the spread of HIV/AIDS. Participation in social organizations, a type of social capital, may provide an important channel to deliver information on safe sex practices and influence beliefs and attitudes towards Sexually Transmitted Infections (STI) prevention, including HIV/AIDS. Leveraging social capital to improve HIV/AIDS prevention may be an important component to address this public health problem of high incidence and prevalence of HIV/AIDS in migrants [4].

Social capital is defined as the power, benefit, value emanating from any social interaction among individuals, groups, institution, communities or networks. It is the intrinsic and extrinsic benefit that members gain for belonging to a group or network. A type of social capital includes the number and types of social organizations. In China, social organizations include governmental organization like various political parties and non-governmental organization include alumni association and cultural & sports clubs. According to 2013 statistics from China, the number of social organizations reached 289,026, which is a 51.2% increase compared with 10 years ago [5]. Increases in social organization participation has played an important role in HIV/AIDS prevention and control and has gradually gained popularity in recent years [6, 7]. For instance, Bhattacharjee et al. investigated the role of membership in peer groups and found that community mobilization and peer group formation reduced HIV-related risk and vulnerability among female sex workers [8]. Campbell et al. explored pathways between community participation and HIV prevention, treatment and impact mitigation in Zimbabwe and found that community group membership is often associated with decreased HIV incedence, improved access to some services particularly amongst women [9]. FSWs who are exposed to social organizations are also at a substantially lower risk of STIs than those who are unexposed [10].

HIV/AIDS knowledge and attitudes towards HIV/AIDS and high-risk behaviors are the measures of the HIV/AIDS prevention effect. The key question is if understanding whether the improvement of knowledge can change migrants’ attitudes towards HIV/AIDS can reduce HIV-related high-risk behaviors. Our previous study found that HIV/STI knowledge is related to attitude and practice for market vendors [11]. Malcolmet et al.’ research showed knowledge alone is not sufficient to eliminate stigmatizing attitudes and behaviors [12, 13]. Lim et al.’s findings suggest that understanding HIV more broadly may be associated with lower HIV-related risk behavior and more comprehensive HIV knowledge may reduce HIV-related high-risk behavior [14].

In this paper, we aimed to explore the role of different types of social organizations on the prevention of HIV/AIDS among migrants. The difference on HIV/AIDS knowledge levels between participants and non-participants for each organization were obtained. The impact of HIV/AIDS knowledge levels on attitudes towards HIV/AIDS and risk behavior was also analyzed for organizations with significant differences between two groups.

## Methods and materials

### Ethical approval

Informed consent is obtained from all the subjects before enrollment in the study. This study is prospectively performed and approved by the Biomedical Ethics Committee, Anhui Medical University with the following reference number: 20110238.

### Studying setting

Anhui province is located in the southeast region of China with relatively low HIV/AIDS prevalence among Chinese provinces. This province has been selected for several reasons as follows: (1) Although with low prevalence, the morbidity showed an increasing trend in recent years. (2) More migrants can be found in Anhui province compared with other provinces with high prevalence. (3) Considering the feasibility, the reason to choose Anhui province is to guarantee the compliance of participates. As the provincial capital, Hefei is chosen for study site because many migrants have poured into this city in recent year. This study was conducted for 3 months from June to September in 2011. Based on the geographic distribution of migrants, we selected four representative areas in Hefei: construction sites, hotels, large markets and large enterprises.

### Study population and data collection

This study examined a convenient selected sample, with the inclusion and exclusion criteria outlined below. To participate in our study, migrants needed to meet the following criteria: (1) ≥18 years old; and (2) people who reside in this city for more than 6 months, but are not generally considered part of the official census count, which meaning that they did not have an official resident permit to live in Hefei. People who travel, study, visit friends and relatives, join the army are not included.

Trained investigators from the Anhui Medical University conducted face-to-face interviews with consented subjects, under the support of staff at the local Center for Disease Control and Prevention (CDC). Most of the data collection was undertaken either in the meeting rooms or other separate places which local CDC provided. With an overall response rate of 92.6%, we conducted full interviews with a total of 758 participants.

### Measures

#### HIV/AIDS KAP survey

**Fig. 1:**
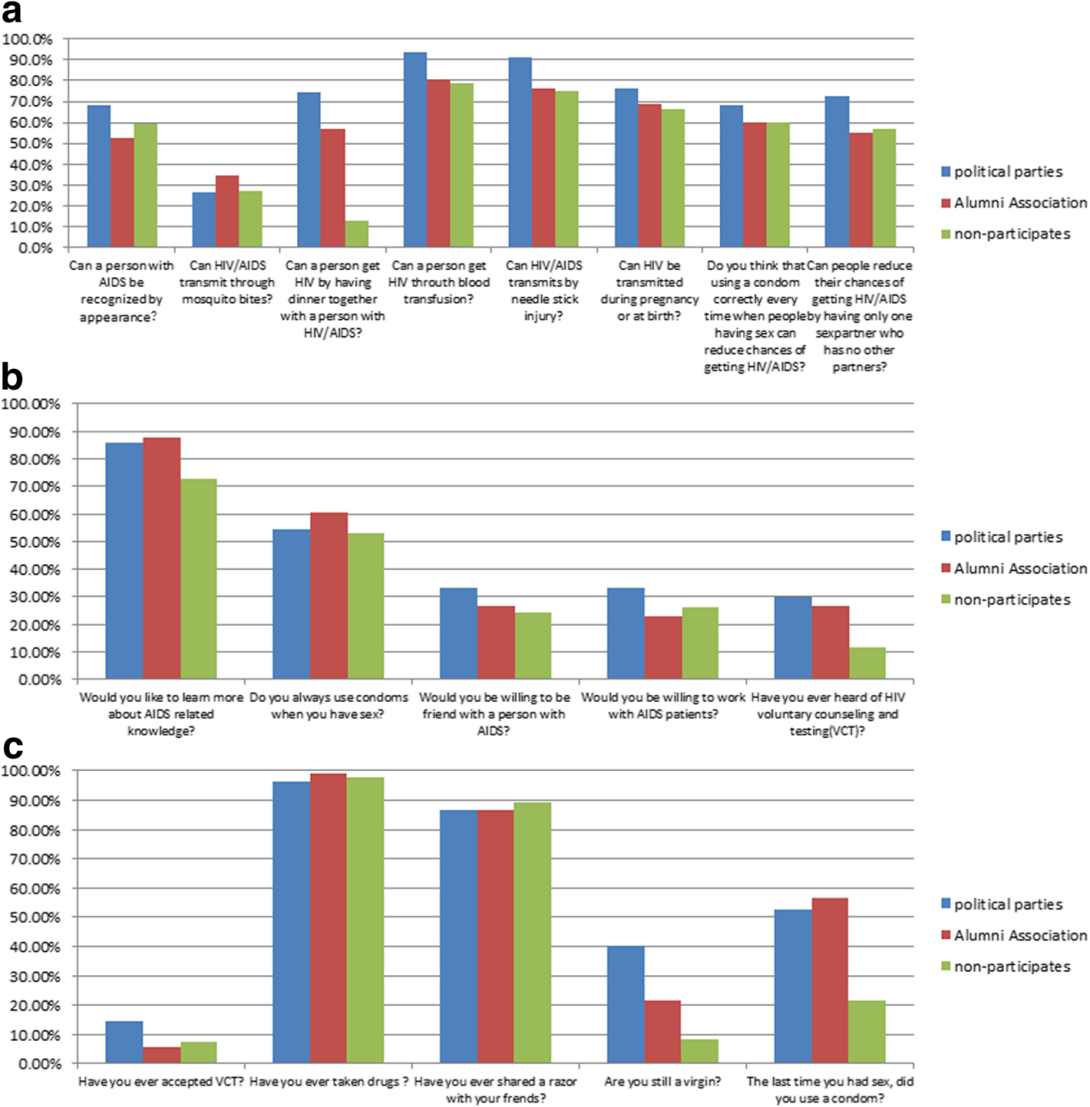
The correct ratio of **a** 8 questions concerning HIV/AIDS knowledge, **b** 5 questions concerning HIV/AIDS attitude, and **c** 5 questions concerning HIV/AIDS risk behaviors among political parties, alumni association and non-participates

The HIV/AIDS KAP survey included questions regarding HIV/AIDS knowledge and attitudes towards HIV/AIDS and HIV-related high-risk behaviors. A total of 18 questions were asked including 8 questions concerning HIV/AIDS knowledge, 5 questions concerning HIV/AIDS attitude and 5 questions concerning HIV/AIDS risk behaviors as illustrated in Fig. 1 shows. The right answers of knowledge, attitude and HIV/AIDS risk behavior are “No, No, No, Yes, Yes, Yes, Yes, Yes”, “Yes, Yes, Yes, Yes, Yes” and “Yes, No, No, Yes, Yes”, respectively. With the right answer, the participant’s attitude and behavior towards a problem are defined as positive attitude and positive behavior, respectively. The negative attitude include not willing to learn HIV/AIDS-related knowledge, not willing to use condom when having sex, discriminating against the HIV/AIDS patients, and not willing to know about VCT. And the positive behaviors include accepting VCT, keeping away from drugs, keeping good habits like not sharing razor, and avoiding high-risk sexual behavior like using condom. Each question was scaled with 3 points. A result recorded of ‘3’ refers to a correct answer, a result of ‘2’ refers to an unclear answer, and a result of ‘1’ represents a wrong answer; 3 = correct; 2 = unclear; 1 = wrong. Subjects with a behavior score of 1 point are defined as high risk behavior. All correct responses are summed, and those who had correct answers (3 points) of HIV/AIDS knowledge for more than 4 questions are considered having a high HIV/AIDS knowledge level. Those who had correct answers (3 points) of HIV/AIDS attitudes and risk behaviors more than 2 questions respectively, were considered having high levels of HIV/AIDS knowledge of attitudes and behaviors.

#### Social support rating scale

The SSRS is a dimension of social capital measured though a self-reported questionnaire, which has been used in our previous studies [15]. It has been proved highly reliable and valid among a wide range of Chinese populations including people who participate in the social organizations.

#### Covariates

The social demographic characteristics included age, marriage, education, hobby, and income. All these variables were categorized and listed in Table 1. Social Organization was classified into 5 groups. Alumni association is defined as peer groups for schoolmates. Political parties included the Communist party and other political parties in Chinese mainland. Cultural & sports clubs are for people with similar hobbies in cultural or sports activities. Other social organization refers to organizations with sample sizes less than 10.

**Table 1:** Social demographic characteristics of migrants, categories are mutually exclusive and exhaustive

	n	Percentage(%)
Gender		
Male	523	69.0
Female	235	31.0
Age Group		
18-20	72	9.5
20-29	307	40.5
30-39	169	22.3
≥ 40	210	27.7
Occupations		
Catering industry workers	134	17.7
Agricultural workers	196	25.8
Enterprise workers	209	27.6
Construction workers	219	28.9
Income		
< 1000	209	27.6
1000-2000	344	45.4
> 2000	205	27.0
Education		
Some or completed primary education	437	57.7
Secondary education	232	30.6
Secondary education or higher	89	11.7
Social Organization		
Alumni association	142	18.7
Political parties	114	15.0
Cultural & sports club	41	5.4
Other social organizations	312	41.2
Non-participates	149	19.6

### Statistical analysis

All data is manually entered into Epidata 3.0 software by trained staff. In order to reduce error rate, the data entry was performed by two persons at the same time by cross-checked, and the verification was accomplished by other two persons who have data processing experience in our research group. Various statistical methods were utilized in the data analysis according to the distribution of the data. A descriptive analysis was performed on the sample, and the results were expressed as means and standard deviations (SDs), frequencies, and percentages. Chi-square tests were performed to assess whether there were significant differences in HIV-related knowledge between participants and non-participants. To test the potential association between HIV/AIDS knowledge and attitudes towards HIV/AIDS and risk behavior, univariate and multivariable logistic regression was employed to calculate crude and adjusted odds ratios (ORs) at the 95% confidence interval (CI). The odds ratios are adjusted by knowledge levels about HIV/AIDS, gender, ages, occupation, individual income and education level. All statistical analysis is performed by SPSS 19.0 version and *P* value < 0.05 was taken as statistically significant.

## Results

### Descriptive statistics

Seven hundred fifty-eight samples are collected with response rate of 92.6% and the mean age of the respondents is 31.9 with a standard deviation of 10.7. The majority of migrants are men (69%), who had a primary education or less (57.7%), and had an individual monthly income below 2000 yuan (~ 300 $). Among the migrants surveyed, 114 people participated in political parties, accounting for 15% of the total. One hundred forty-two people participated in alumni associations, accounting for 18.7% of the total. Forty-one migrants participated in various types of cultural and sports clubs, accounting for 5.4% of the total. A full descriptive summary of the respondents is provided in Table 1.

### Chi-square test

HIV/AIDS knowledge, attitudes and HIV-related high-risk behaviors of migrants have been investigated and more details were shown in Fig. 1. According to the result, it is found that the majority of migrants are not willing to accept the HIV voluntary counseling testing.

Findings presented on Table 2 show that a majority (71.6%) of political party members had more HIV/AIDS knowledge, while 68.4% of non-political party members had less HIV/AIDS knowledge. In addition, people who participated in alumni associations (76.8%) had more HIV/AIDS knowledge than people who do not belong to the alumni association (67.9%). We observed a statistically significant relationship between membership in political parties (χ^2^ = 15.266, *P*-value< 0.001) and alumni associations (χ^2^ = 4.32, P-value = 0.043) with high HIV/AIDS knowledge.

**Table 2:** Distribution of participation in social organizations and AIDS knowledge score

Social organizations		Low knowledge	High knowledge	Total n	χ^2^	p-value
Political parties	Participation	21 (28.4%)	93 (71.6%)	114	9.20	0.002
	No participation	210 (31.6%)	434 (68.4%)	644		
	Total	231 (30.0%)	527 (70.0%)	758		
Alumni association	Participation	33 (23.2%)	109 (76.8%)	142	4.32	0.043
	No participation	198 (32.1%)	418 (67.9%)	616		
	Total	231 (30.0%)	527 (70.0%)	758		
Cultural & sports club	Participation	7 (17.0%)	34 (83.0%)	41	3.67	0.057
	No participation	224 (31.2%)	493 (68.8%)	717		
	Total	231(30.0%)	527 (70.0%)	758		

### Logistic regression analysis

#### Political parties

In Table 3, the multinomial logistic regression showed that HIV/AIDS knowledge was significantly associated with attitudes towards HIV/AIDS (OR = 0.19, 95% CI: 0.06-0.66). HIV/AIDS knowledge was significantly related to HIV-related high-risk behaviors (OR = 3.49, 95% CI: 1.22-9.99), after adjustment for age, occupation, individual income and education level. Positive attitude towards HIV/AIDS in the high HIV/AIDS knowledge group was 0.194 times higher than that in lower knowledge group. HIV/AIDS knowledge group and positive behaviors in high HIV/AIDS knowledge group was 3.486 times higher than that in the less HIV/AIDS knowledge group. These results indicated that HIV/AIDS knowledge is positively correlated with positive behaviors and negatively correlated with attitudes towards HIV/AIDS. Education levels are also associated with attitudes towards HIV/AIDS (OR = 5.7, 95% CI: 1.33-24.40). In addition, having a high income (family monthly income> 2000 Yuan) (OR = 4.895, 95% CI: 1.18-20.37) and being agricultural workers (OR = 6.389, 95% CI: 1.03-39.62) are significantly associated with an increasing odds of positive behaviors.

**Table 3:** Associations between AIDS knowledge and attitudes by political party

Variables		Sample size	High attitude score				High behavior score			
			cOR^a^ (95%CI)	P	aOR^b^ (95%CI)	P	cOR (95%CI)	P	aOR (95%CI)	P
Knowledge levels about AIDS	Low	21	1	–	1	–	1	–	1	–
	High	93	***0.25 (0.09-0.71)***	***0.009***	***0.19 (0.058-0.66)***	***0.007***	***2.90 (1.06-7.84)***	***0.037***	***3.49 (1.21-9.99)***	***0.02***
Gender	male	90	1	–	1	–	1	–	1	–
	female	24	0.854 (0.34-2.12)	0.734	0.943 (0.29-3.04)	0.922	0.26 (0.10-0.69)	0.007	0.475 (0.14-1.57)	0.224
Ages	20<	19	1	–	1	–	1	–	1	–
	20-29	70	1.01 (0.29-3.9)	0.987	0.69 (0.08-5.75)	0.733	1.80 (0.50-6.46)	0.367	0.67 (0.07-5.85)	0.719
	30-39	4	1.144 (0.43-3.04)	0.788	1.326 (0.22-7.93)	0.757	2.0 (0.72-5.55)	0.183	1.08 (0.15-7.8)	0.934
	≥40	21	2.73 (0.24-30.66)	0.416	2.05 (0.11-38.31)	0.631	0.68 (0.58-7.6)	0.744	3.03 (0.17-54.39)	0.452
Occupation	Industry worker	29	1	–	1	–	1	–	1	–
	Agricultural worker	4	1.42 (0.50-3.96)	0.507	3.19 (0.57-17.62)	0.183	***8.94 (2.7-29.41)***	***0.000***	***6.39 (1.03-39.62)***	***0.046***
	Enterprise workers	51	3.00 (0.28-32.09)	0.364	2.32 (0.14-38.40)	0.555	0.78 (0.07-8.52)	0.837	0.50 (0.04-7.70)	0.605
	Construction workers	30	1.22 (0.49-3.00)	0.67	2.68 (0.55-13.07)	0.224	1.39 (0.53-3.63)	0.508	1.13 (0.21-6.01)	0.883
Individual income	1000 <	23	1	–	1	–	1	–	1	–
	1000-2000	69	1.91 (0.56-6.46)	0.301	1.96 (0.44-8.66)	0.374	2.89 (0.74-11.37)	0.128	2.98 (0.55-15.99)	0.204
	> 2000	22	0.86 (0.3-2.34)	0.755	0.61 (0.17-2.24)	0.461	***5.86 (1.79-19.10)***	***0.003***	***4.89 (1.17-20.37)***	***0.029***
Education level	Some or completed primary education	22	1	–	1	–	1	–	1	–
	Secondary education	55	2.53 (0.81-7.88)	0.111	***5.70 (1.33-24.40)***	***0.019***	0.66 (0.22-1.90)	0.438	0.95 (0.19-4.80)	0.953
	Above secondary education	37	0.98 (0.43-2.26)	0.967	1.74 (0.61-4.89)	0.297	0.73 (0.32-1.96)	0.433	0.90 (0.30-2.70)	0.856

^a^Crude odds ratio

^b^Adjusted odds ratio

All items with *P* < 0.05 were bold italics

#### Alumni association

As shown in Table 4, the multivariate adjusted ORs indicated that high HIV/AIDS knowledge is significantly associated with a lower odds of positive attitudes towards HIV/AIDS (OR = 0.20, 95% CI: 0.07-0.61). HIV/AIDS knowledge is not significantly related to positive HIV-related behaviors. Those in the high HIV/AIDS knowledge group are less likely to have positive attitudes towards HIV/AIDS compared to those in the lower HIV/AIDS knowledge group (OR: 0.20, 95% CI: 0.08-0.61). In addition, being agricultural workers is significantly associated with an increased likelihood of positive behaviors (OR = 7.82, 95%CI: 2.05-29.77).

**Table 4:** Associations between AIDS knowledge and attitudes by alumni association

Variables		Sample size	High attitude score				High behavior score			
			cOR^a^ (95%CI)	P	aOR^b^ (95%CI)	P	cOR (95%CI)	P	aOR (95%CI)	P
Knowledge levels about AIDS	Low	33	1	–	1	–	1	–	1	–
	High	109	***0.16 (0.06-0.44)***	***0.000***	***0.20 (0.08-0.61)***	***0.005***	2.00 (0.92-4.36)	0.079	1.56 (0.63-3.85)	0.334
Gender	male	92	1	–	1	–	1	–	1	–
	female	40	1.913 (0.87-4.22)	0.109	0.317 (0.21-1.67)	0.586	0.764 (0.36-1.62)	0.483	1.06 (0.41-2.80)	0.905
Ages	20<	33	1	–	1	–	1	–	1	–
	20-29	79	3.23 (0.34-31.07)	0.306	4.25 (0.33-55.15)	0.268	5.31 (0.56-50.53)	0.146	2.20 (0.18-24.36)	0.547
	30-39	21	3.33 (0.37-29.96)	0.286	3.17 (0.28-35.20)	0.347	3.52 (0.72-5.55)	0.261	1.31 (0.13-13.63)	0.809
	≥40	9	5.63 (0.53-58.90)	0.15	5.73 (0.45-72.33)	0.177	1.53 (0.13-17.33)	0.727	1.22 (0.09-15.25)	0.877
Occupations	Catering industry worker	40	1	–	1	–	1	–	1	–
	Agricultural worker	17	0.28 (0.09-0.80)	0.018	0.35 (0.57-17.62)	0.111	8.80 (2.71-28.53)	0.000	***7.81 (2.05-29.77)***	***0.003***
	Enterprise workers	54	0.41(0.10-1.67)	0.215	0.39 (0.08-1.95)	0.252	1.32 (0.23-6.63)	0.732	1.88 (0.33-10.50)	0.475
	Construction workers	31	0.93(0.36-2.35)	0.876	1.17 (0.54-13.07)	0.792	2.53 (0.82-7.78)	0.105	2.73 (0.21-6.01)	0.124
Individual income	1000<	39	1	–	1	–	1	–	1	–
	1000-2000	73	0.85 (0.31-2.55)	0.903	1.03 (0.28-3.73)	0.966	1.21 (0.73-11.37)	0.729	0.59 (0.16-2.16)	0.43
	> 2000	30	0.28 (0.24-1.50)	0.599	0.75 (0.25-2.24)	0.61	1.75 (0.67-4.57)	0.251	1.09 (0.37-3.18)	0.87
Education level	Some or completed primary education	38	1	–	1	–	1	–	1	–
	secondary education	74	0.29 (0.09-0.86)	0.027	0.30 (0.08-1.12)	0.075	0.52 (0.18-1.51)	0.232	0.38 (0.1-1.38)	0.139
	Above secondary education	30	0.66 (0.43-2.26)	0.319	0.48 (0.15-1.51)	0.213	0.66 (0.27-1.62)	0.364	0.58 (0.18-1.81)	0.347

^a^Crude odds ratio

^b^Adjusted odds ratio

All items with *P* < 0.05 were bold italics

## Discussion

Our study provides an initial exploration of correlations between membership in social organizations and migrants’ HIV/AIDS knowledge, attitudes and HIV-related high-risk behavior. With further development, our findings can be used to develop evidence-based policy to improve programs for HIV/AIDS prevention and control for migrant populations in China.

As can be seen, a relatively low proportion of migrants participated in social organizations. This phenomenon may due to the essence of migrants. Compared with the city’s permanent population, the income of migrants is lower. They have to spend more time on work. Therefore, most migrants have no spare time to join the social organizations.

According to the results, migrants who are members of political parties or alumni associations have more HIV/AIDS knowledge than those who do not participate in any social organizations. Social organizations have promoted the advertisement of HIV/AIDS knowledge. In recent years, the government has paid more attention to social organizations so that HIV/AIDS-related knowledge can be spread more extensively among migrants, highlighting the need to take advantage of social organizations to carry out various training courses, which include HIV/AIDS prevention and knowledge. However, misconceptions about the routes of transmission are common. According to the answers by respondents, there are some false beliefs that HIV/AIDS can be transmitted by mosquito bites, a belief that has also been observed in previous studies [16]. To resolve this problem, social organizations should educate migrants on the routes of HIV/AIDS transmission and common fallacies about the virus.

The results of multiple logistic regression analysis demonstrate that HIV/AIDS knowledge is an important factor that influences political party members’ behaviors and attitudes towards HIV/AIDS. High knowledge levels may have positive effects on people’s behaviors. Research has shown that health education of HIV/AIDS can improve peoples’ understanding of HIV/AIDS, thus it could decrease high-risk behaviors [17, 18]. The impact of high knowledge level on HIV/AIDS-related behavior is not significant for migrants who participated in alumni associations. Different roles played by different social organizations may explain this phenomenon. Alumni associations or NGOs may have insufficient capacities, be deficient in resources, and not systematic enough compared with political parties to effectively reduce HIV/AIDS-related behavior [19, 20]. On the other hand, mobility of migrants causes difficulties for HIV/AIDS-related education and intervention services. Most of the migrants are from suburbs, and those who have lower HIV/AIDS-related knowledge than long-term residents are more likely to be engaged in high-risk behaviors [21]. Our previous research has shown that migrants are more likely to have high risk HIV-related sexual behavior than non-migrants [22]. Fang et al. mentioned that even if HIV/AIDS universal education improved knowledge levels that it will be hard to change their attitudes toward HIV/AIDS [23–25]. Most migrants have positive behaviors towards drug abuse regardless of social organization participation. In China, it is well known that taking drugs is harmful, requiring lots of money and with serious legal punishment, which makes people consciously regulate their own behaviors. On the one hand, humanistic care could be developed through community support and psychological care for migrants such as organizing some healthy, informative, interesting entertainment activities. With a rich amateur life, prostitution and drug abuse are obviously reduced while loneliness and dissatisfaction are also reduced and confidence is established in life. On the other hand, multi-channel peer education and behavioral interventions could be carried out by social organizations. Health publicity and bulletin boards could be made and free condoms could be distributed in high HIV/AIDS epidemic areas. Social organizations with abundant resources could make in-depth publicity and face to face education for high-risk HIV populations.

There are still substantial intolerant attitudes towards HIV positive and HIV/AIDS patients. Interestingly, although migrants who participate in either political parties or alumni associations had higher-levels of HIV/AIDS knowledge and are willing to obtain more HIV-related knowledge. However, they also had more negative attitudes toward HIV/AIDS than migrants with low-levels of HIV/AIDS knowledge, especially in the aspect of accepting HIV/AIDS patients. Other studies suggest that improving HIV/AIDS knowledge is not enough for to change attitudes. Yu et al.’s study which collected articles published between 1996 and 2006 concerning the effect of HIV/AIDS education intervention in migrants shows that health education for preventing HIV/AIDS is effective in changing attitudes in migrants [26]. However, Chen et al.’s study which is based on a sample of nurses in a single hospital in Jiamusi City, Heilongjiang, China, suggests the opposite conclusion. In their study, nurses who have a better understanding of HIV/AIDS prevention are more likely to have negative attitudes toward HIV/AIDS [27]. But study by Anahita Tavoosi also shows although the knowledge level seems to be moderately high, misconceptions about the routes of transmission are common among Iranian students [28]. So if we only disseminate preventive knowledge and promote a positive attitude, the results are likely to be unsatisfactory. The main reason for negative attitudes is that most of migrants have less education which affects their understanding and acceptance of HIV/AIDS knowledge. Better understanding of HIV/AIDS knowledge actually increased people’s panic. They fear for their personal safety, their family members’ health and others’ attitudes towards them so that they want to stay away from this sensitive issue [27]. We also found that the majority of migrants are not willing to accept the HIV voluntary counseling testing (VCT). Two main factors led to this phenomenon. First, migrants cannot fully understand VCT. Second, social discrimination and stigma make people fear to seek and accept testing. Worrying about exposing their privacy is also a reason. This result shows that it is important for migrants to eliminate the misunderstanding so as to change their attitudes. Social organizations also should be more comprehensive when disseminating HIV/AIDS knowledge, not only to enhance the awareness of HIV/AIDS, but also to deepen the understanding of transmission and non-transmission routes to decrease panic and discrimination against HIV/AIDS. Social organizations can also take measures, including cooperating with local health sector, setting up VCT consulting point convenient to migrants, training VCT services consultant to improve the quality of their service, persisting in the principle of protecting consultants’ privacy and perfecting care service after counseling and testing, to change migrants’ attitude toward VCT and increase utilization of VCT in migrants. For migrants who have been infected with HIV/AIDS, it is applicable and effective for social organizations to take their advantages, which included training peer educators, developing civil relief, helping migrants infected with HIV/AIDS to engage in production to support themselves and providing psychological counseling for them, their participation can make services more professional, comprehensive and sustainable.

Here we observe a significant association between Agricultural workers and positive behavior (*P* < 0.05), but a wide range for 95% confidence interval. This phenomenon may due to the small sample size. Migrant who had higher incomes tended to exhibit more positive behaviors. People with higher incomes may have more educated social circles, which allows them receive more informative knowledge of HIV/AIDS [29–31].

Limitations of this study include the use of data from a cross-sectional survey, where causality cannot be ascertained. In addition, our sample size is not big enough due to limited funding. Finally, the results may not be generalized to all Chinese migrants. Our data is collected in Anhui province, which has a relatively low HIV/AIDS prevalence for China, and thus may not reflect the situation in other provinces due to regional differences in the epidemic characteristics of HIV/AIDS, prevention and control measures, funding, and policy environment.

## Conclusion

Overall, community groups appear to raise awareness of HIV/AIDS-related knowledge. Therefore, increasing the percentage of migrants who are members of community groups could help increase HIV/AIDS knowledge. Although the knowledge is transferred into positive behavior, the two groups show different abilities. Due to excellent organization capacity, scale and the advantages in resources, the party organization could transfer knowledge into positive behavior better than alumni association as a government agency. It also indicates that organizations that target migrants specifically could have a much larger impact in HIV/AIDS knowledge on this relatively clandestine and high-risk population. As HIV/AIDS knowledge publicity is not comprehensive, migrants with high knowledge on HIV/AIDS would have more negative attitudes towards HIV/AIDS than that with low-levels of HIV/AIDS knowledge. Although the current situation there is not complete, social organizations still play an important positive role on HIV/AIDS prevention for migrants. They present great potential in curbing the spread of HIV/AIDS. With the completing of organizational mechanism, social organizations will make a greater contribution to HIV/AIDS prevention for migrants in the future.

## Abbreviations

CI: Confidence interval
KAP: Knowledge, Attitude and Practice
NGOs: Non-Government Organizations
OR: Odds ratio
VCT: Voluntary counseling testing
